# Characteristics of Sluggish Cognitive Tempo among adults with ADHD: objective neurocognitive measures align with self-report of executive function

**DOI:** 10.3389/frcha.2023.1188901

**Published:** 2023-07-24

**Authors:** Beth Krone, Lenard A. Adler, Deepti Anbarasan, Terry Leon, Richard Gallagher, Pooja Patel, Stephen V. Faraone, Jeffrey H. Newcorn

**Affiliations:** ^1^ Department of Psychiatry, Icahn School of Medicine at Mount Sinai (ISMMS), New York, NY, United States; ^2^ Department of Psychiatry, New York University (NYU) Grossman School of Medicine, New York, NY, United States; ^3^ Norton College of Medicine, SUNY Upstate Medical University, Syracuse, NY, United States

**Keywords:** Attention Deficit Hyperactivity Disorder (ADHD), Sluggish Cognitive Tempo (SCT), Cognitive Disengagement Syndrome (CDS), Adult ADHD, Executive Function, Neurocognitive Phenotyping, Cambridge Neuropsychological Test Automated Battery (CANTAB), Barkley Adult SCT Scale (BAARS)

## Abstract

**Introduction:**

Sluggish Cognitive Tempo (SCT) is a syndrome characterized by cognitive hypo-arousal that often appears as daytime sleepiness or drowsiness, mental fogginess, being easily confused, having difficulty with holding and manipulating information in working memory, and being forgetful. Although it frequently co-travels with attention-deficit/hyperactivity disorder (ADHD) or other conditions and confers significantly greater impairment, there are few studies examining SCT among adults with ADHD. Understanding what features SCT confers in association with ADHD, distinct from other conditions associating with ADHD, is critically important to confirm if SCT is a distinct syndrome that requires special assessment methods and special, distinct treatment efforts to reduce its impact. This study describes the clinical and neuropsychological features of SCT in a sample of adults with well-defined ADHD, and examines the relationship of SCT with other measures of ADHD, neurocognition, executive function (EF), and impairment.

**Methods:**

A sample of n = 106 adults with ADHD, ages 18-57 years, was assessed for SCT using the Barkley SCT scale. Adults with (SCT+) and without (SCT-) SCT received a comprehensive clinical assessment battery, and neuropsychological testing. Clinical and neuropsychological variables were examined for their associations with SCT. The variables were treated with Principal Axis Factoring with Promax with Kaiser Normalization to elucidate latent constructs and determine performance profiles associated with SCT among people with ADHD.

**Results:**

EF Deficits and emotional dyscontrol (ED) symptoms significantly differentiated adults with ADHD and SCT whether measured via self or clinician report. Additionally, significantly greater impairment via both clinician and participant report was seen in the SCT + versus SCT - cohorts. SCT was also associated with a significantly distinct profile on the neuropsychological battery, characterized by a pattern of slower latencies and cognitive strategy choices across CANTAB and WAIS subtests, that reveals difficulty with increased cognitive load, which primarily accounted for the higher level of impairment in the SCT group.

**Discussion:**

The convergence of clinical ratings and neurocognitive measures of EF deficits is consistent with the conclusion that SCT represents a distinct subgroup of adults with ADHD.

## Introduction

Sluggish Cognitive Tempo (SCT) describes a set of symptoms that includes daydreaming, trouble initiating tasks, under-motivation, under-arousal, mental confusion, and being slow-moving (1, 2). In a large population survey, Barkley posited nine core symptoms of SCT as follows: (1) prone to daydreaming instead of concentrating; (2) trouble staying alert/awake in boring situations; (3) being easily confused; (4) being easily bored; (5) feeling spacey/in a fog; (6) frequently feeling lethargic; (7) being underactive/having less energy than others; (8) being slow-moving; and (9) not processing information quickly/accurately. Barkley identified adults as having SCT if they had five or more of the nine symptoms scored as “often” or “very often” on the SCT subscale of the Barkley Adult ADHD Rating Scale-IV: Self-Report (3) (BAARS-IV). The syndrome has recently been referred to as cognitive disengagement syndrome (4), but for consistency with earlier literature, we will refer to it as SCT throughout this manuscript.

SCT is common in adults; Barkley found a population rate of 5.8% and SCT frequently co-occurs with ADHD (about half of the Barkley sample) as well as anxiety, depressive, and learning disorders (1, 2, 5). SCT can also be significantly impairing (6). Lunsford-Avery et al. found that increasing SCT severity was associated with multiple aspects of impairment (e.g., work, social relations, and community activities) as reported by self or collateral ratings, even after controlling for ADHD ratings (7).

Both ADHD and SCT have been referred to as disorders of executive function (EF) [e.g., studies by Sergeant (8) and Barkley (5)]. Decades of research using objective neuropsychological assessment indicate that measurable aspects of EF often disrupted in ADHD include impulse control (measured through response inhibition tasks), planning and strategy (measured through tower/stocking and maze tasks), cognitive flexibility and control (measured through set-shifting and maintenance tasks), focus (measured through interference control), and working memory (measurable through a variety of tasks, such as the n-back). Several rating scales have been developed to quantify these EF deficits by capturing reports of the frequency and intensity of the behaviors through which they are thought to manifest. Difficulty withholding a response on a neuropsychological go/no-go task and reports of “frequently acting before thinking” are related (though not perfectly associated) constructs of impulse control. With this in mind, the clinical descriptors of SCT, which include being easily confused, having difficulty with maintaining attentional arousal, and processing information quickly and accurately, suggest SCT describes EF difficulties distinct from those in ADHD. The research suggests this is the case among children with ADHD + SCT, but there is very little research examining EF in relation to ADHD + SCT among adults. In the few prior studies of adults with ADHD + SCT, SCT was associated with EF deficits in problem-solving and organization (9, 10) and in global EF (1, 2, 9–13). Even when accounting for both ADHD severity and co-morbid anxiety, SCT remained independently associated with EF deficits and emotional self-control (2, 9–11). Stimulant treatment attenuated these effects, though it was uncertain whether the attenuation represented indirect effects from treating ADHD or from some unspecified direct treatment effect on SCT (11). Another study examining the multimodal treatment of adults with ADHD + SCT also showed these adults benefit from currently approved ADHD medications and psychotherapies tailored to EF deficits (14). In our interim analysis of the current study, our group reported SCT associated with more severe ADHD inattention, as well as with greater deficits in self-report but not clinician-rated EF (15), making an objective neurocognitive assessment of EF all the more salient for evaluating the association of SCT with EF in the context of ADHD.

As there are currently few randomized clinical trials that evaluate the response of SCT symptoms to pharmacotherapy in adults, we undertook a two-site study [NYU Grossman School of Medicine (NYU) and Icahn School of Medicine at Mount Sinai (ISMMS)] in adults with ADHD to examine the phenomenology of SCT (contrasting ADHD with and without SCT) and the responsiveness of SCT symptoms to the stimulant lisdexamfetamine vs. placebo in the SCT-positive (SCT+) adults. We previously reported the results of the treatment trial in adults with SCT and ADHD (16) and an interim analysis of the baseline ADHD sample, comparing clinical measures in the SCT+ versus SCT-negative (SCT−) cohorts in 87 participants in the NYU sample (15). The interim analysis found that adults with ADHD and SCT had greater symptoms of inattention and ratings of impairment than those with ADHD but without SCT. The cohort with both disorders also had higher ratings of EF deficits on the BRIEF-A, but not on clinical ratings assessed via the expanded ADHD symptom rating scale. However, the interim analysis did not have enough power in the SCT+ cohort to address these inconsistencies between the BRIEF-A and expanded ADHD symptom scales, and also control for potential effects of ADHD symptoms on differentiating the presence of SCT. In this manuscript, we report the rating scale findings in a larger sample, with an expanded SCT+ cohort, while also investigating neuropsychological test patterns [Wechsler Adult Intelligence Scale-IV (WAIS) and The Cambridge Neuropsychological Test Automated Battery (CANTAB)]. This allows a fuller understanding of the clinical presentation and neuropsychological profile of adults with ADHD and SCT.

## Methods

**Figure 1:**
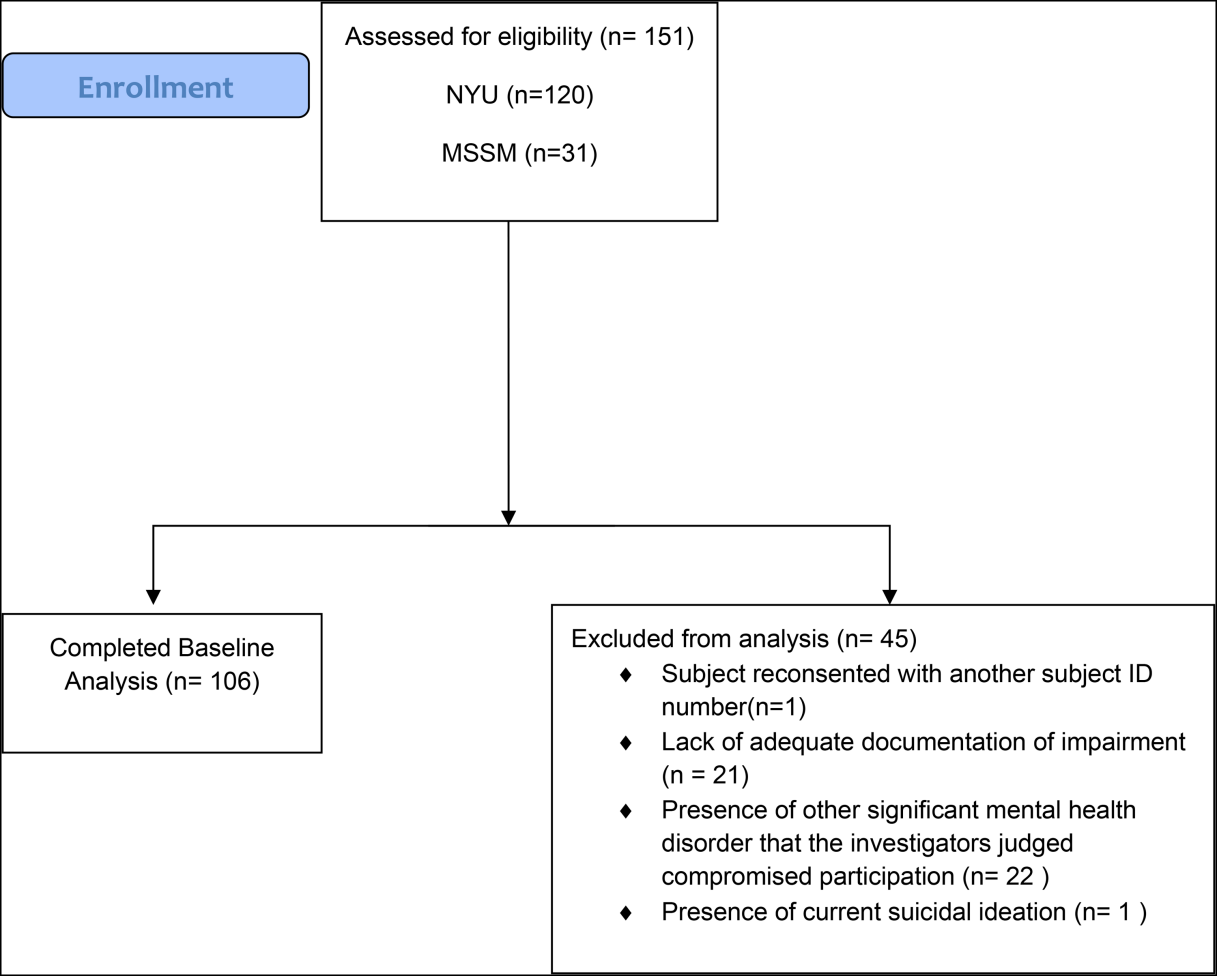
Patient disposition.

In total, 151 adults with ADHD were consented (NYU: *n* = 120; ISMMS: *n* = 31), with 106 adults with ADHD completing this baseline investigation (see Figure 1 for participant disposition) (NYU: *n* = 87, 48 SCT+, 39 SCT−; ISMMS: *n* = 19, 15 SCT+, 4 SCT−), of which 63 were SCT+ and 43 were SCT−.

### Assessments

Participants were administered the expanded Adult ADHD Clinical Diagnostic Scale (ACDS) v1.2 to ascertain the presence of DSM-5 adult ADHD, the Adult ADHD Investigator Symptom Rating Scale (AISRS) expanded version to assess ADHD symptoms, the Mini International Neuropsychiatric Interview 7.0 (MINI) to establish DSM-5 disorders, the Behavior Rating Inventory of Executive Function-Adult Version (BRIEF-A) to measure executive functioning, the Barkley Functional Impairment (BFIS) and Clinical Global Impression (CGI) scales to measure impairment, and the Barkley SCT Scale (3) to measure the severity of SCT. Suicidality was assessed using the Columbia-Suicide Severity Rating Scale (17). Medical and psychiatric histories, as well as demographics, were self-reported.

All clinical assessments were administered in person by board-certified psychiatrists, a clinical psychologist, or a research nurse, each with over 10 years of experience in adult ADHD research. All clinicians were trained per standard procedures before the initiation of the study (18). All diagnostic or clinical questions were discussed with the two site principal investigators, both experts in adult ADHD (LA and JN).

### Inclusion and exclusion criteria

The inclusion criteria were as follows: (1) adults aged 18–60 years meeting the DSM-5 criteria for a primary diagnosis of inattentive or combined type ADHD as diagnosed via ACDS v1.2; (2) demonstrated significant impairment, based on the norms of the BFIS; (3) the SCT+ cohort needed to have ≥5 items on the Barkley SCT Scale rated 3 (“often”) or 4 (“very often”) as well as a total SCT symptom score of ≥26 (4) and a T-score of ≥65 on the Metacognition Index Subscale of BRIEF-A; and (4) the SCT− cohort had <5 items on the Barkley SCT Scale rated 3 (“often”) or 4 (“very often”) and a total SCT symptom score <26.

Participants were excluded for the following reasons: (1) they met the DSM-5 criteria for a primary diagnosis of hyperactive-impulsive presentation ADHD as diagnosed via the ACDS v1.2; (2) they had any other current psychiatric disorder, determined via the MINI, that necessitated pharmacotherapy; (3) they had current suicidal ideation or a history of suicide attempts; (4) they had a lifetime history of bipolar disorder or any psychotic disorder (as per the MINI); (5) they were female and planning to become pregnant or were currently breast-feeding; (6) they had a positive urine drug toxicology at baseline; (7) they had any significant clinical issues that, in the opinion of the investigator, would prevent the person from participating in the study or compromise the participant's safety; and (8) they had previously used lisdexamfetamine (as this was the study drug during the treatment phase of the protocol).

The study was approved by the Institutional Review Boards at these institutions and registered at ClinicalTrials.gov (identifier: NCT02635035). The trial was conducted between January 2016 and April 2018.

### Rating scales

#### ACDS v1.2

A diagnosis of ADHD was evaluated via v1.2 of the ACDS (11, 12), a semi-structured diagnostic interview that is extensively used in adult ADHD investigations (13, 14) (19). The ACDS v1.2 clinical interview first assesses childhood symptoms of ADHD and then an expanded set of recent (past year) adult ADHD symptoms, including all DSM-5 criteria A1 and A2 symptoms. The scale includes developmentally appropriate prompts and stems questions with modified language designed to capture DSM symptoms of ADHD as they present in childhood and adulthood. After asking all prompts and probes for each symptom, the clinician rates the symptom severity as 1 (never), 2 (mild), 3 (moderate), or 4 (severe).

The ACDS v1.2 has been expanded to include 13 additional symptoms that query executive function deficits (EFDs; 9 items) and emotional dyscontrol (ED; 4 items). EFDs contain symptoms of higher-level cognitive processes of the organization, planning, keeping things in mind (working memory), task initiation, and planning. ED includes behavioral descriptors of changeable mood, irritability, and emotional over-reactivity. Specific prompts and probes have been included for the 13 additional ACDS v1.2 EFD and ED items to help guide the rater in assessing these accessory symptoms in adulthood.

#### Adult ADHD investigator rating scale

At baseline, ACDS v1.2 scores were converted to AISRS scores to measure ADHD symptom severity (as has been done in prior adult ADHD trials), after verifying the similarity of symptoms in the recent past versus in the last year (20, 21). Both the ACDS v1.2 and AISRS use the same prompts and a 4-point scale (“none,” “mild,” “moderate,” and “severe”; the latter two being the cutoff for clinical impairment for each item). Previous studies have established the rationale for this transformation, which is centered on the high agreement between AISRS and ACDS v1.2 scores and the need to minimize participant burden. Therefore, all subsequent discussion of ADHD symptom rating data will refer to these scores on the expanded 31-item AISRS scale.

The AISRS provides an expanded ADHD symptom score (31 items), as well as inattentive (IA) and hyperactive-impulsive (H-I) subscale scores (9 items each). The total ADHD symptom score on the AISRS is the sum of the IA and H-I subscales (18 items), hereafter referred to as the AISRS total symptom score. As noted, the EFD subscale has nine items and the ED subscale has four items. Both the 18-item and the expanded version of the AISRS have been extensively used to evaluate baseline symptoms in both cross-sectional and treatment studies.

#### Behavior rating inventory of executive function—adult version (BRIEF-A)

The BRIEF-A is a norm-referenced, self-report scale of EF with T-scores ≥65 indicating clinically significant impairment for indices and composite scores (22, 23). Nine clinical subscales measure behaviors associated with EF. The first five assess the ability to initiate behaviors (initiate), use working memory, plan and mentally organize or structure behaviors (plan/organize), judge progress on a task (task monitor), and organize objects and materials for use in tasks (organization of material); these five items refer to cognitive manifestations of symptoms. These items also contribute [together with other items (see below)] to a Metacognition Index (MI). The next four subscales [to inhibit behaviors (inhibit), to flexibly shift between tasks (shift), to control emotions (emotional control), and to monitor and regulate behaviors to adapt to situational or task demands (self-monitor)] refer to the ability to regulate behavioral responses. These items contribute to a Behavioral Regulation Index (BRI). The Global Executive Complex (GEC) score, comprising the various subscales described above, is considered a global EF measure.

#### MINI 7.0

The MINI was used to evaluate psychiatric co-morbidity (24). The MINI is a structured clinical interview used to assess DSM-5 psychiatric disorders and has been widely used to evaluate psychiatric co-morbidity in adult ADHD studies and in trials involving other mental health disorders.

#### Barkley Functional Impairment Scale (BFIS)

The BFIS is a self-report measure of perceived current impairment in 15 different major life activities rated on a 10-point Likert scale (0 = not impaired, 9 = severely impaired) (25). The activity domains include interactions at home, handling personal affairs, completing chores, activities, and work, social interactions, and relationships with friends and family. Mean ratings can be calculated to quantify impairment. The scale has been normed in a large epidemiologic survey of individuals with and without ADHD.

#### CGI-severity (CGI-S)

Overall impairment was assessed by the CGI-S scale, a widely used clinician-rated measure of global ADHD impairment that uses a 7-point Likert rating (1 = normal, 7 = among the most severely ill patients) (26).

#### Barkley SCT scale

Adult SCT was assessed using the SCT subscale of the BAARS-IV (3). This is a nine-item self-report that is scored on a 3-point rating scale (1 = “never or rarely,” 2 = “sometimes,” 3 = “often,” and “very often”). The Barkley scale cut-score thresholds are based on a large (*N* = 1,249) normative sample. Responses of “often” or “very often” are considered clinically relevant manifestations of SCT behaviors. The presence of four (of nine) clinically relevant items places a respondent over the 93rd percentile and in the “mildly symptomatic” range. To ensure the presence of SCT, we used the more conservative presence of five items and a cut-score of 26, which places a respondent over the 95th or 96th percentile, and in the “moderately symptomatic” range, which is above Barkley's suggested cutoff of 93rd percentile and symptom counts. The scale has been shown to have good psychometric properties, including test-retest reliability (1). The Cronbach's alpha of the Barkley SCT scale was 0.84 for this sample, which was almost identical to the 0.83 for the NYU cohort reported in the interim analyses.

### Neurocognitive measures

#### Wechsler Adult Intelligence Scale-IV (WAIS-IV)

The WAIS-IV is a standardized assessment of cognitive abilities (27). In this study, the Processing Speed Index (PSI), comprising the Coding (Cd) and Symbol Search (SS) subtests, was used to determine the speed and accuracy with which participants visually discriminate simple geometric symbols and produce manual (written) responses. Where the SS subtest requires minimal visual scanning and motor movement (drawing a line through a symbol), the cognitive (learning to associate a number with a symbol) and motor requirements (writing a number) are much greater for the Cd subtest.

#### The Cambridge Neuropsychological Test Automated Battery (CANTAB)

The CANTAB is a standardized, direct measure of neurocognitive function and frontal lobe dysfunction (28, 29). Tasks comprising the ADHD battery are normed with clinical thresholds and target attentional processes regulated via the prefrontal cortex. The CANTAB ADHD battery has been used to assess dopaminergic and noradrenergic pathways in ADHD treatment response and to characterize EF weaknesses among adults with ADHD (30). However, to date, there have been no studies using the CANTAB to assess SCT. The CANTAB tasks used in this study were as follows: (1) the Motor Screening Task (MOT), which discriminates between sensorimotor deficits and lack of comprehension by measuring the participant's speed of response and accuracy of pointing at the correct target; (2) the Spatial Working Memory (SWM), which assesses retention and manipulation of visuospatial information and provides measures of performance for executive function (e.g., cognitive load), strategy (e.g., the efficiency of strategies used), and errors (e.g., incorrect responses); (3) the Rapid Visual Information Processing (RVP) measures sustained visual attention and arousal, providing measures of latency, probability of false alarms, and sensitivity; (4) the Stop Signal Task (SST) assesses response inhibition by providing reaction times for “go” and “stop” signals, and the proportion of successful inhibited responses. The task also provides a measure of direction errors, since choice objects may appear either on the right or left side of the screen. The Attention Switching Task was added to the battery for this study, though it is not part of the standard CANTAB ADHD battery. This task provides a direct measure of EF as cognitive control of attention, providing a measure of attention shifting.

### Data collection, preparation, and analysis

#### Clinical variables: SCT, ADHD, impairment BRIEF major indices ratings

Data were analyzed via logistic regressions to examine the variables that differentiated the SCT+ and SCT− cohorts. Initial analyses examined potential demographic differences, including sex, age, ethnicity, and race. Significant demographic predictors were then used as covariates in the analyses, examining the following variables as potential predictors of SCT: Barkley SCT score, AISRS IA, AISRS H-I, AISRS EFD, ASISRS ED; Brief GEC T-; Brief MCI T-; CGI-S; and BFIS MI scores. A pre-hoc decision was made to include the site as a covariate given the preferential recruitment of SCT+ participants at the ISMMS site. The analysis of ADHD symptoms in the SCT+ and SCT− groups uses the ADHD-RS with prompts total score (IA and H-A) as it reflects more recent symptoms. The AISRS total score (IA and H-I) is used as a covariate in these analyses. This avoids duplicating the analyses of the potential effects of two 18-item ADHD scales in differentiating between the SCT cohorts and distinguishing between acute and chronic symptoms.

The potential differences for the neuropsychological tests were examined, as noted below, via two-tailed *t*-tests, Mann–Whitney *U* tests, and factor analyses.

Variable distributions were examined for outliers, skew, and kurtosis. The distributions were relatively normal, although there were several somewhat kurtotic clinical-scale variables, as should be expected in a dataset consisting of only clinical cases (no healthy controls). The minor kurtosis did not need to be addressed, because the intended principal axis factoring (PAF) is robust to these violations of distribution (31).

A descriptive characterization of participants classified as with or without SCT was conducted. The neurocognitive differences between individuals with and without SCT were examined with two-tailed *t*-tests and Mann–Whitney *U* tests.

Dimension reduction was accomplished with PAF with Promax rotation (see Supplementary Text). PAF provides information about the way in which neurocognitive performance variables and clinical scales factor into SCT while respecting the structure of latent variables. The Promax rotation allows for the factors to be correlated and provides unique and shared variance estimates, as should be expected. This is desirable given that the variables are multiple measures of the same psychological constructs. In using this oblique rotation method, we first examined the pattern matrix for information about the item and factor loadings, and then the factor correlation matrix to assess correlations between the factors.

## Results

Sample demographics are presented in Table 1. Most of the sample met the criteria for the combined ADHD presentation. The mean age of the whole sample was 31.9 ± 8.6 years, with the SCT+ cohort being slightly older (33.0 ± 9.7 years vs. 30.2 ± 7.1 years. Of the sample, 36.8% were men, with the percentage being somewhat lower in the SCT+ group (33.3% v. 41.86%). The sample was 50.0% (*n* = 53) white, 13.2% (*n* = 14) black, 18.9% (*n* = 20) Hispanic, 16.0% (*n* = 17) Asian, and 1.9% (*n* = 2) other/unknown. Table 1 shows the breakdown by SCT status. There was a greater proportion of white participants (65.1%, *n* = 28) in the SCT− sample compared to the SCT+ sample (39.7%, *n* = 25). Nine participants in the NYU cohort had a lifetime mood or anxiety disorder. Six participants in the ISMMS cohort had a lifetime mood or anxiety disorder. No participant met the criteria for a current (within the past year) mood or anxiety disorder.

**Table 1:** Sample demographics by SCT phenotype.

	Total sample (*n* = 106)	ADHD + SCT (*n* = 63)	ADHD-SCT (*n* = 43)
Age (years)	31.9 ± 8.8	33.0 ± 9.7	30.2 ± 7.1
Male	36.8	33.33	41.86
Female	63.2	66.67	58.14
Race/ethnicity			
Non-Hispanic white		39.68	65.12
Non-Hispanic black		19.05	4.65
Hispanic		20.63	16.28
American Indian or Alaskan		0	0
Asian		19.05	11.63
Other or Unknown		1.59	2.33

ADHD, attention deficit/hyperactive disorder; SCT, Sluggish Cognitive Tempo.

Values are given as % or mean ± SD.

### Clinical variables: comparing samples of patients with and without SCT

Age was a significant predictor of SCT. This was not surprising given that the SCT+ cohort was somewhat older (×2 (1) = 3.94, *p* = 0.047); the site was also a significant predictor of SCT, which was also expected, as the higher proportion of SCT+ participants in the ISMMS cohort was *pre-hoc* established (×2 (1) = 4.28, *p* = 0.039). The other demographic variables were not significant (NS; sex: ×2 (1) = 0.71, NS; ethnicity: ×2 (1) = 0.01, NS; race: ×2 (1) = 1.89, NS). Therefore, the logistic regressions for the clinical variables were adjusted for the two statistically significant demographic variables (site and age) (Table 2).

**Table 2:** Logistic regressions predicting SCT, adjusting for site and age.

	Sample	ADHD + SCT	ADHD-SCT			
	*N* = 106	*N =* 63	*N =* 43			
	*M ± SD*	*M ± SD*	*M ± SD*	*df*	*X^2^*	*p*
Barkley SCT^a^	25.7 ± 5.8	29.7 ± 3.1	19.9 ± 3.5	3	16.41	<0.000
ADHD-RS Total^b^	37.9 ± 8.2	39.2 ± 7.2	35.9 ± 9.3	3	16.41	<0.000
AISRS IA^c^	21.0 ± 3.5	21.7 ± 2.8	19.9 ± 4.1	3	21.34	<0.000
AISRS H-I^d^	16.0 ± 6.0	16.0 ± 5.9	15.8 ± 6.1	3	13.02	<0.005
AISRS EFD^e^	22.5 ± 5.0	23.5 ± 4.6	21.0 ± 5.2	3	19.88	<0.000
AISRS ED^f^	5.6 ± 3.4	6.4 ± 2.8	4.3 ± 3.8	3	18.42	<0.000
BRIEF-A GEC^g^	75.1 ± 10.1	79.5 ± 8.3	68.6 ± 8.9	3	46.6	<0.000
BRIEF-A MCI^h^	78.5 ± 9.7	82.7 ± 7.9	72.4 ± 9.0	3	46.58	<0.000
BFIS MCI^i^	5.6 ± 1.3	6.1 ± 1.1	4.9 ± 1.4	3	39.4	<0.000
CGI-S^j^	4.7 ± 0.6	4.8 ± 0.6	4.5 ± 0.6	3	16.2	<0.000

ADHD, attention deficit/hyperactive disorder; SCT, Sluggish Cognitive Tempo.

^a^ Barkley Sluggish Cognitive Tempo Scale.

^b^ Attention Deficit Hyperactivity Disorder Rating Scale Total Score.

^c^ AISRS Inattention Scale.

^d^ AISRS Hyperactivity/Impulsivity Scale.

^e^ AISRS Executive Functioning Disorder Subscale.

^f^ AISRS Emotional Dysregulation Subscale.

^g^ BRIEF-A Global Executive Composite.

^h^ BRIEF-A MetaCognition Index.

^i^ Barkley Functional Impairment Scale MetaCognition Index.

^j^ Clinical Global Impairment-Severity.

ADHD symptoms (AISRS subscales), associated EF and ED symptoms (AISRS expanded subscales), BRIEF-A measures of EF (GEC and MCI), and measures of impairment (CGI and BFIS) all significantly discriminated SCT+ from SCT− participants. These differences were due to SCT associating with higher levels of ADHD, EF, and ED symptoms and impairment. Not surprisingly, the Barkley SCT Scale significantly predicted differences between the SCT+ and SCT− cohorts, with the SCT+ sample having substantially higher scores.

To examine the potential contribution of ADHD scores to differences between the SCT+ and SCT− groups, logistic regressions were conducted adjusting for total AISRS scores (IA + H-I), in addition to age and site. These analyses found that EF deficits and ED symptoms (as elicited on AISRS subsets and the BRIEF-A) still differentiated the SCT+ and SCT− groups, as did the Barkley SCT scores. The SCT cohort maintained significantly greater impairment on both the CGI and BFIS (Table 3) after correcting for total ADHD symptoms. Thus, the significantly greater impairment seen in the SCT+ cohort was not accounted for by their higher ADHD scores. Rather, the greater impairment seen in participants with SCT was due to the presence of EFD and possibly also ED. The AISRS IA and H-I subscales were not significantly different between the SCT+ and SCT− cohorts.

**Table 3:** Logistic regressions predicting SCT, adjusting for ADHD severity, site, and age.

	Sample	ADHD + SCT	ADHD-SCT			
	*N =* 106	*N =* 63	*N =* 43			
	*M ± SD*	*M ± SD*	*M ± SD*	*df*	*X^2^*	*p*
Barkley SCT^a^	25.7 ± 5.8	29.7 ± 3.1	19.9 ± 3.5	2	111.47	<.000
AISRS IA^b^	21.0 ± 3.5	21.7 ± 2.8	19.9 ± 4.1	2	5.47	NS
AISRS H-I^c^	16.0 ± 6.0	16.0 ± 5.9	15.8 ± 6.1	2	3.70	NS
AISRS EFD^d^	22.5 ± 5.0	23.5 ± 4.6	21.0 ± 5.2	2	8.44	.015
AISRS ED^e^	5.6 ± 3.4	6.4 ± 2.8	4.3 ± 3.8	2	11.23	<.000
BRIEF-A GEC^f^	75.1 ± 10.1	79.5 ± 8.3	68.6 ± 8.9	2	40.33	<.000
BRIEF-A MCI^g^	78.5 ± 9.7	82.7 ± 7.9	72.4 ± 9.0	2	30.33	<.000
BFIS MCI^h^	5.6 ± 1.3	6.1 ± 1.1	4.9 ± 1.4	2	24.57	<.000
CGI-S^i^	4.7 ± 0.6	4.8 ± 0.6	4.5 ± 0.6	2	60.8	.048

ADHD, attention deficit/hyperactive disorder; SCT, Sluggish Cognitive Tempo.

^a^ Barkley Sluggish Cognitive Tempo Scale.

^b^ AISRS Inattention Scale.

^c^ AISRS Hyperactivity/Impulsivity Scale.

^d^ AISRS Executive Functioning Disorder Subscale.

^e^ AISRS Emotional Dysregulation Subscale.

^f^ BRIEF-A Global Executive Composite.

^g^ BRIEF-A MetaCognition Index.

^h^ Barkley Functional Impairment Scale MetaCognition Index.

^i^ Clinical Global Impairment-Severity.

### Neuropsychological measures

Of the 104 participants who completed valid CANTAB MOT and SST subtests, there were no significant differences between those with and without SCT in the speed of motor skills without cognitive load (MOT Mean Latency). Nor was there a difference in their ability to perform the task correctly without a cognitive load (MOT Mean Errors). There were no significant differences in any of the measures of response inhibition with minimal cognitive load (Table 4).

**Table 4:** Clinical and neuropsychological assessments, differences ADHD + SCT and ADHD-SCT.

		*N*	M	SD	*F*	Sig.	*t*	*df*	*p*	MD	SE	95% CI	
BRIEF Inhibit	SCT+	63	70.81	11.433	0.934	0.336	2.155	104	**0** **.** **033**	4.996	2.318	0.399	9.592
	SCT−	43	65.81	12.127									
BRIEF Shift	SCT+	63	70.37	11.266	0.003	0.960	5.093	104	**0**.**000**	11.202	2.199	6.841	15.564
	SCT−	43	59.16	10.898									
BRIEF Emotional Control	SCT+	63	62.56	12.706	0.003	0.956	3.751	104	**0**.**000**	9.230	2.461	4.350	14.110
	SCT−	43	53.33	12.037									
BRIEF Self-Monitor	SCT+	63	63.46	14.022	2.814	0.096	2.867	104	**0**.**005**	7.344	2.561	2.265	12.423
	SCT−	43	56.12	11.179									
BRIEF Initiate	SCT+	63	77.56	9.303	1.324	0.253	5.776	104	**0**.**000**	10.044	1.739	6.596	13.492
	SCT−	43	67.51	7.974									
BRIEF Working Memory	SCT+	63	82.78	9.037	1.157	0.285	4.158	104	**0**.**000**	7.661	1.843	4.007	11.316
	SCT−	43	75.12	9.713									
BRIEF Plan/Org	SCT+	63	79.54	9.483	1.558	0.215	4.459	104	**0**.**000**	9.075	2.035	5.039	13.110
	SCT−	43	70.47	11.371									
BRIEF Task Monitor	SCT+	63	80.97	9.105	2.606	0.110	4.254	104	**0**.**000**	8.410	1.977	4.490	12.331
	SCT−	43	72.56	11.179									
BRIEF Organize Material	SCT+	63	68.65	10.955	0.724	0.397	2.679	104	**0**.**009**	5.953	2.222	1.547	10.360
	SCT−	43	62.70	11.632									
WAIS Symbol Search -SS	SCT+	61	9.64	3.540	4.220	0.043	−2.024	99	**0**.**046**	−1.286	0.635	−2.546	−0.025
	SCT−	40	10.93	2.336									
WAIS Coding -SS	SCT+	61	10.30	2.996	0.315	0.576	−1.988	98	**0**.**050**	−1.166	0.587	−2.331	−0.002
	SCT−	39	11.46	2.634									
WAIS Processing Speed (PSI)	SCT+	61	99.48	16.495	1.990	0.161	−2.274	98	**0**.**025**	−6.909	3.039	−12.94	−0.879
	SCT−	39	106.38	11.704									
AST Congruency cost (M, correct)	SCT+	60	59.159	55.517	0.581	0.448	1.648	101	0.102	18.399	11.16	−3.743	40.540
	SCT−	43	40.760	56.342									
AST Switching cost (M, correct)	SCT+	60	261.488	137.513	0.113	0.738	0.215	101	0.830	6.233	28.924	−51.144	63.610
	SCT−	43	255.255	154.364									
AST M correct latency	SCT+	60	603.142	131.012	1.318	0.254	2.117	101	**0**.**037**	51.361	24.260	3.236	99.486
	SCT−	43	551.781	106.490									
AST M correct latency (congruent)	SCT+	60	576.416	126.681	1.671	0.199	1.870	101	*0*.*064*	44.139	23.601	−2.679	90.957
	SCT−	43	532.277	104.918									
AST M correct latency (incongruent)	SCT+	60	634.673	140.828	0.765	0.384	2.529	101	**0**.**013**	68.606	27.123	14.801	122.411
	SCT−	43	566.067	128.270									
AST M correct latency (blocks 3,5) [non-switching blocks]	SCT+	60	472.529	104.120	1.400	0.239	2.695	101	**0**.**008**	50.144	18.603	13.240	87.0478
	SCT−	43	422.385	74.948									
AST M correct latency (block 7) [switching block]	SCT+	60	735.680	184.511	0.195	0.660	1.458	101	0.148	53.366	36.610	−19.259	125.992
	SCT−	43	682.313	181.420									
AST % correct trials	SCT+	60	92.688	6.473	4.578	0.035	−1.938	101	*0*.*055*	−2.239	1.155	−4.532	0.052
	SCT−	43	94.927	4.646									
MOT M latency	SCT+	61	829.321	290.419	0.037	0.847	1.257	102	0.212	81.379	64.760	−47.073	209.831
	SCT−	43	747.942	369.319									
MOT M error	SCT+	61	9.593	3.455	0.358	0.551	−0.939	102	0.350	−0.647	0.689	−2.016	0.720
	SCT−	43	10.241	3.479									
RVP A’	SCT+	61	0.888	0.060	3.302	0.072	−2.468	102	**0**.**015**	−0.027	0.011	−0.049	−0.005
	SCT−	43	0.915	0.048									
RVP Probability of hit	SCT+	61	1.829	9.657	2.578	0.111	0.783	102	0.435	1.1,554	1.4,750	−1.7,703	4.0,811
	SCT−	43	0.673	0.177									
RVP Total false alarms	SCT+	61	9.902	26.267	10.003	0.002	1.854	102	*0*.*067*	7.4,830	4.0,363	−0.5,230	15.4,891
	SCT−	43	2.419	3.500									
RVP M latency	SCT+	61	523.802	127.678	10.562	0.002	2.865	102	**0**.**005**	62.791	21.915	19.321	106.261
	SCT−	43	461.011	78.300									
SST SSRT (last half)	SCT+	60	227.179	75.118	3.197	0.077	1.801	101	*0*.*075*	23.551	13.078	−2.3,928	49.4,948
	SCT−	43	203.628	48.745									
SST M correct RT on GO trials	SCT+	60	516.332	121.652	0.129	0.720	1.034	101	0.303	26.119	25.248	−23.967	76.2,050
	SCT−	43	490.213	132.702									
SST Median correct RT on GO trials	SCT+	60	480.383	123.175	0.080	0.778	0.811	101	0.419	20.953	25.844	−30.315	72.2,218
	SCT−	43	459.430	137.553									
SST Direction errors on stop-and-go trials	SCT+	60	2.683	3.437	3.180	0.078	1.113	101	0.268	0.683	0.614	−0.534	1.901
	SCT−	43	2.000	2.469									
SST Proportion of successful stops (last half)	SCT+	60	0.481	0.103	0.140	0.709	−0.830	101	0.409	−0.017	0.0,208	−0.0,586	0.0,241
	SCT−	43	0.498	0.106									
SST SSD (50%) (last half)	SCT+	60	252.703	146.166	0.033	0.857	−0.157	101	0.876	−4.494	28.640	−61.308	52.3,195
	SCT−	43	257.197	139.272									
SWM Between errors	SCT+	61	18.38	10.431	0.001	0.973	2.776	102	**0**.**007**	5.703	2.055	1.627	9.778
	SCT−	43	12.67	10.155									
SWM Strategy	SCT+	61	17.43	3.528	0.869	0.353	3.908	102	**0**.**000**	2.822	0.722	1.389	4.254
	SCT−	43	14.60	3.762									

ADHD, attention deficit/hyperactive disorder; AST, attention switching task; MOT, Motor Screening Task; RVP, Rapid Visual Information Processing; SCT, Sluggish Cognitive Tempo; SST, Stop Signal Task; SWM, Spatial Working Memory; WAIS, Wechsler Adult Intelligence Scale.

Bold = statistically significant at *p* < .05.

Among participants with valid RVP subtest scores on the CANTAB (*N* = 104), those with SCT detected fewer targets [RVP A’: SCT+ = 0.888 ± 0.060; SCT− = 0.915 ± 0.048; t(102) = −2.468; *p* = 0.015]. They also took longer to make decisions and respond [RVP mean latency: SCT+ = 503.80 ± 127.67; SCT− = 461.01 ± 78.9; t(102) = 2.865; *p* = 0.005].

Among the participants who completed the WAIS subtests to provide a measure of processing speed (*N* = 101), overall processing speed (PSI) was significantly slower among participants with SCT (*N* = 61, mean = 99.48 ± 16.49) than without SCT (*N* = 40, mean = 106.38 ± 11.70; t(98) = −2.274; *p* = 0.02). On the Symbol Search Subtest, participants with SCT (mean = 9.64 ± 3.54) demonstrated a significantly slower speed for visually scanning a page and matching novel geometric designs [t(99) = −2.024; *p* = 0.046] compared to the participants without SCT (mean = 10.93 ± 2.33). On the Coding subtest, the participants with SCT (mean = 10.30 ± 2.99) trended slower in their ability to associate a simple geometric symbol with numbers, and to draw the shape when prompted by the number [t(99) = −1.988; *p* = 0.05] than those without SCT (mean = 11.46 ± 2.63).

Among the 103 participants with valid AST subtest scores on the CANTAB, the measures that were significantly different indicated that participants with SCT paid a higher cognitive cost when discriminating between stimuli [AST Congruency Cost Median: SCT+ mean = 63.10 ± 60.84; SCT− mean = 36.80 ± 48.42; *t*(101) = 2.35; *p* = 0.021]. They took longer to make correct responses [AST mean correct latency: SCT+ = 603.14 ± 131.01; SCT− = 551.78 ± 106.49; t(101) = 2.117; *p* = 0.037]. They took longer to correctly respond to incongruent stimuli [AST mean correct latency (incongruent): SCT+ = 634.67 ± 140.82; SCT− = 566.06 ± 128.87; t(101) = 2.529; *p* = 0.013 (mean correct latency incongruent, Mann–Whitney U test, *p* = 0.031)]. They spent more time determining correct responses when task stimuli did not switch [AST mean correct latency (blocks 3,5) (non-switching blocks): SCT+ = 472.52 ± 140.12; SCT− = 422.38 ± 74.98; t(101) = 2.695; *p* = 0.008 (*p* = 0.042)]. There was also a trend for a lower proportion of trials correctly completed [AST percent correct trials: SCT+ mean = 92.68 ± 6.47; SCT− mean = 94.92 ± 4.64; t(101) = −1.938; *p* = 0.055].

Participants with SCT performed significantly more poorly on the CANTAB SWM subtest, using a less efficient cognitive strategy [SCT+ mean = 17.43 ± 3.52; SCT− mean = 14.60 ± 3.76; t(102) = 3.908; *p* = 0.000]. They also made significantly more errors [SCT+ mean = 18.38 ± 10.43; SCT− mean = 12.67 ± 10.15; t(102) = 2.776; *p* = 0.007].

The planned Forced 5 Factor model explained 49.83% of the variance in SCT; however, the factor correlation matrix (Table 5) revealed a very strong correlation between factors 4 and 1, and the proportion of variance explained dropped precipitously between factors 3 and 4 and remained reasonably and consistently low with subsequent factors. As such, we interpreted the more conservative Forced 3 Factor model that explained 41.23% of the variance (Tables 6–8). The factor correlation matrix indicated that Factors 1 and 3 were moderately correlated (r = 0.372), and Factors 2 and 3 were moderately correlated (r = 0.327) as would be expected with related traits. However, Factors 1 and 2 shared very little overlap (r = 0.102) and an examination of the items comprising the factors explains why. The pattern matrix is displayed in Table 6, the pattern structure matrix is displayed in Table 7, and the unrotated matrix is displayed in Table 8. Items with factor loadings above 0.320 are shown.

**Table 5:** Factor correlation matrix.

Factor	1	2	3
1	1.000	0.102	0.372
2	0.102	1.000	0.327
3	0.372	0.327	1.000

Extraction method: principal axis factoring; rotation method: Promax with Kaiser normalization.

**Table 6:** Three-factor pattern matrix^a^ (β).

	Factor		
	1 (SCT: EF + ED)	2 (ED)	3 (Distractible)
Plan Organize	0.817		
Initiate	0.813		
Working Memory	0.743		
Task Monitor	0.692		
AISRS (EFD)	0.674		
AISRS (IA)	0.627		
Inhibit	0.592		
Organize Material	0.567		
Emotional Control	0.547		
Self-Monitor	0.526		
Shift	0.521		
AISRS (ED)	0.503		
SCT (BAARS)	0.482		
AISRS (HI)	0.364		
RVP Probability of hit			
AST Mean correct latency (block 7) (switching block)		0.914	
AST Mean correct latency		0.906	
AST Mean correct latency (congruent)		0.873	
AST Mean correct latency (incongruent)		0.857	
AST Switching cost (Mean, correct)		0.711	
SST SSD (50%) (last half)		0.705	−0.419
SST Mean correct RT on GO trials		0.656	
SST Proportion of successful stops (last half)		0.443	
AST Congruency cost (Mean, correct)			
Composite Score-Processing Speed			−0.821
Symbol Search-Scaled Score			−0.717
Coding-Scaled Score			−0.675
SST SSRT (last half)			0.622
RVP A’			−0.618
AST Percent correct trials			−0.538
RVP Mean latency			0.487
SWM Between errors			0.476
MOT Mean latency			0.404
RVP Total false alarms			0.323

ADHD, attention deficit/hyperactive disorder; AST, attention switching task; ED, emotional dyscontrol; EF, executive function; MOT, Motor Screening Task; RVP, Rapid Visual Information Processing; SCT, Sluggish Cognitive Tempo; SST, Stop Signal Task; SWM, Spatial Working Memory; WAIS, Wechsler Adult Intelligence Scale.

Extraction method: principal axis factoring; rotation method: Promax with Kaiser normalization. RVP A’ (probability of detecting targets) higher = better. Factor 1 had an Eigenvalue of 7.5 and accounted for 20.9% of the variance. It was labeled the SCT Factor and showed the associations between SCT, the clinical variables, and EF measures. This was the only factor to include SCT as a predictor on the pattern matrix. In all, there were 14 strong components in this grouping. The remaining components were EF problems as reported on all nine subscales of the BRIEF-A; EF problems, ED problems, Hyperactivity/Impulsivity, and Inattention as rated by clinicians on all four domains of the AISRS. Factor 2 had an Eigenvalue of 4.7 and accounted for 13.1% of the variance. It was labeled the Executive Function (EF) Factor. There were eight strong components in this grouping: four variables measuring longer response latencies for all task conditions (congruent cue, incongruent cue, and switching block) of the CANTAB AST, a variable reflecting increased cognitive burden during tasks that require mental flexibility (switching cost) on the AST, and three variables of the CANTAB SST task that represent the cognitive burden during response inhibition (faster response times when inhibition is not required (on “go” tasks) within the context of greater variability in response times indicates a higher cognitive cost of inhibition responses). Factor 3 had an Eigenvalue of 2.6 and accounted for 7.2% of the Variance. It was labeled the Distractibility Factor. Twelve components had strong or moderate loadings on this factor. It is notable that the loading for AISRS ED was 0.000 for this factor on the pattern matrix compared to weak but non-zero loadings on the other two. The items most strongly contributing to this factor were negative associations (meaning better) WAIS-5 PSI and performance on its component scales. Other contributors were: longer RTs on the CANTAB SST, with less variability in performance over time (SST SSD); better detectability, but more false alarms, and longer latencies on the CANTAB RVP; longer latencies on the CANTAB MOT; lower percent correct trials on the CANTAB AST; and worse performance on the CANTAB Working Memory task.

^a^ Rotation converged in five iterations.

AST congruency cost + = faster congruent trials, − = faster incongruent trials. AST switch cost, + = faster non-switch trials, − = faster switch trials. AST percent correct higher = better. SWM Between Errors, higher = worse (more repetitive incorrect selection).

**Table 7:** Three-factor structure matrix (zero-order correlations).

	Factor		
	1 (SCT: EF + ED)	2 (EF)	3 (Distractible)
Plan_Org	0.808		
Initiate	0.798		
Working Memory	0.764		0.334
Task Monitor	0.705		
Inhibit	0.619		
AISRS (EFD)	0.617		
Shift	0.612		0.435
SCT (BAARS)	0.591		0.474
Self-Monitor	0.579		0.328
Organize Material	0.571		
Emotional Control	0.547		
AISRS (IA)	0.529		
AISRS (ED)	0.507		
AISRS (HI)			
RVP Probability of hit			
AST Mean correct latency (blocks 3,5) (non-switching blocks)			
AST Mean correct latency		0.956	0.454
AST Mean correct latency (block 7) (switching block)		0.951	0.415
AST Mean correct latency (incongruent)		0.921	0.484
AST Mean correct latency (congruent)		0.917	0.421
AST Switching cost (Mean, correct)		0.720	
SST Mean correct RT on GO trials		0.630	
SST SSD (50%) (last half)		0.560	
SST Proportion of successful stops (last half)		0.349	
Composite Score -Processing Speed		−0.382	−0.795
Coding -Scaled Score		−0.403	−0.685
Symbol Search -Scaled Score			−0.669
RVP A’			−0.606
SST SSRT (last half)	0.321		0.596
RVP Mean latency			0.527
AST Percent correct trials			−0.515
SWM Between errors	0.342		0.510
MOT Mean latency			0.383
RVP Total false alarms			

ADHD, attention deficit/hyperactive disorder; AST, attention switching task; ED, emotional dyscontrol; EF, executive function; MOT, Motor Screening Task; RVP, Rapid Visual Information Processing; SCT, Sluggish Cognitive Tempo; SST, Stop Signal Task; SWM, Spatial Working Memory; WAIS, Wechsler Adult Intelligence Scale.

Extraction method: principal axis factoring; rotation method: Promax with Kaiser normalization. RVP A’ (probability of detecting targets) higher = better. A review of the structure matrix supported the interpretation of Factor 1 and Factor 2 from the pattern matrix and identified expanded items for Factor 3 that account for the moderate correlations between Factors 1 and 3, and 2 and 3. Factor 3 shares latency loadings with Factor 2. Factor 3 also shares SCT and three measures of EF (Working Memory, Shift, and Self-Monitor) with Factor 1. On the structure matrix, Factor 1 also picked up the loading for CANTAB Working Memory.

AST congruency cost + = faster congruent trials, − = faster incongruent trials. AST switch cost, + = faster non-switch trials, − = faster switch trials. AST percent correct higher = better. SWM Between Errors, higher = worse (more repetitive incorrect selection).

**Table 8:** Three-factor matrix^a^ (r—unrotated).

	Factor		
	1 (SCT: EF + ED)	2 (EF)	3 (Distractible)
AST Mean correct latency (incongruent)	0.752	−0.554	
AST Mean correct latency	0.712	−0.635	
AST Mean correct latency (block 7) (switching block)	0.675	−0.652	
AST Mean correct latency (congruent)	0.656	−0.634	
Working Memory	0.614	0.424	
SCT (BAARS)	0.602		
Initiate	0.600	0.459	
Plan Organize	0.580	0.523	
Composite Score-Processing Speed	−0.562		0.549
Task Monitor	0.552	0.408	
Organize Material	0.539		
Shift	0.535	0.377	
Inhibit	0.524	0.314	
Coding -Scaled Score	−0.523		0.425
RVP Mean latency	0.446		
RVP A’	−0.444		0.413
SWM Between errors^e^	0.433		
AISRS (ED)	0.398		
Emotional Control	0.392	0.364	
AST Congruency cost (Mean, correct)^b^	0.308		
SST SSD (50%) (last half)		−0.545	0.446
AST Switching cost (Mean, correct)^c^	0.459	−0.527	
SST Mean correct RT on GO trials	0.336	−0.492	
Self-Monitor	0.428	0.441	
AISRS (EFD)	0.377	0.437	
SST Proportion of successful stops (last half)		−0.334	
Symbol Search-Scaled Score	−0.444		0.497
SST SSRT (last half)	0.426		−0.433
AISRS (IA)	0.333		0.402
AST Percent correct trials^d^	−0.364		0.369

ADHD, attention deficit/hyperactive disorder; AST, XX; ED, emotional dyscontrol; EF, executive function; MOT, Motor Screening Task; RVP, Rapid Visual Information Processing; SCT, Sluggish Cognitive Tempo; SST, Stop Signal Task; SWM, Spatial Working Memory; WAIS, Wechsler Adult Intelligence Scale.

Extraction method: principal axis factoring. RVP A’ (probability of detecting targets) higher = better. Although this was an exploratory analysis with a relatively small sample, we were unable to retain all factors with Eigenvalue greater than 1. Retention of factors based on the Eigenvalue is an over-inclusive method that retains noise, and due to the high communality between the measures, we were required to constrain the parameters in order to extract meaningful factors. To do this, we examined the accompanying scree plot to find the natural break point. This point was arguably between 3 and 5, so in following Geert van den Berg (31), we examined the 5 factor's Eigenvalues and contribution to the variance, and we determined that the true break point was after factor 3. The resulting 3 factor pattern (containing the coefficients for the linear combination of the variables) and structure matrices of the PAF were determined to be stable, containing 5 or more contributing items (Costello & Osborne, 2005). Factor 1 had an Eigenvalue of 7.5 and accounted for 20.9% of the variance. It was labeled the SCT Factor and showed the associations between SCT, the clinical variables, and EF measures. This was the only factor to include SCT as a predictor on the Pattern Matrix. In all, there were 14 strong components in this grouping. The remaining components were EF problems as reported on all nine subscales of the BRIEF-A; EF problems, ED problems, Hyperactivity/Impulsivity, and Inattention as rated by clinicians on all four domains of the AISRS. Factor 2 had an Eigenvalue of 4.7 and accounted for 13.1% of the variance. It was labeled the Executive Function (EF) Factor. There were 8 strong components in this grouping: four variables measuring longer response latencies for all task conditions (congruent cue, incongruent cue, and switching block) of the CANTAB AST, a variable reflecting increased cognitive burden during tasks that require mental flexibility (switching cost) on the AST, and three variables of the CANTAB SST task that represent the cognitive burden during response inhibition (faster response times when inhibition is not required (on “go” tasks) within the context of greater variability in response times indicates a higher cognitive cost of inhibition responses). Factor 3 had an Eigenvalue of 2.6 and accounted for 7.2% of the Variance. It was labeled the Distractibility Factor. Twelve components had strong or moderate loadings on this factor. It is notable that the loading for AISRS ED was .000 for this factor on the Pattern Matrix, as compared to weak but non-zero loadings on the other two. The items most strongly contributing to this factor were negative associations (meaning better) WAIS-5 PSI and performance on its component scales. Other contributors were: longer RTs on the CANTAB SST, with less variability in performance over time (SST SSD); better detectability, but more false alarms, and longer latencies on the CANTAB RVP: longer latencies on the CANTAB MOT: lower percent correct trials on the CANTAB AST: and worse performance on the CANTAB Working Memory task. Review of the Structure Matrix supported interpretation of the Factor1 and Factor 2 from the Pattern Matrix, and identified expanded items for Factor 3 that account for the moderate correlations between factors 1 and 3, and 2 and 3. Factor 3 shares latency loadings with Factor 2. Factor 3 also shares SCT and three measures of EF (Working Memory, Shift, and Self-Monitor) with Factor 1. On the Structure Matrix, Factor 1 also picked up the loading for CANTAB Working Memory.

^a^ Three factors extracted. Six iterations required.

^b^ AST congruency cost + = faster congruent trials, − = faster incongruent trials.

^c^ AST switch cost, + = faster non-switch trials, − = faster switch trials.

^d^ AST percent correct higher = better.

^e^ SWM Between Errors, higher = worse (more repetitive incorrect selection).

Each of the three factors represented a separate latent construct (see Supplementary Text). Factor 1 comprised clinical and neurocognitive measures associated specifically with SCT on the BAARS. Factor 2 represented executive function problems characterized by poor cognitive flexibility, worse response inhibition, and worse performance with an increased cognitive burden. Factor 3 represented better processing speed, poor task performance (more errors), and more problems with working memory.

## Discussion

The current finding, compatible across both the clinical ratings and neurocognitive assessments, shows that SCT represents a distinct subset within ADHD and that individuals with ADHD + SCT have higher ratings and neuropsychological measures suggestive of EF deficits, and higher impairment due to the EF deficits (since those were not explained by ADHD severity). Importantly, contrary to the preliminary findings presented in the interim analysis, this held true regardless of whether EF problems were measured via self or clinician report.

The observation of greater impairment in adults with ADHD and SCT vs. those with ADHD alone has been reported previously (1) and was also seen in our interim report (15). All ADHD symptom sets (IA, H-I, EFD, and ED) on the BRIEF and ADHD symptom scales were seen to be significantly higher in the SCT+ cohort in these analyses of the full baseline cohort. When a smaller number of SCT+ participants were analyzed in the interim analysis, similar findings were seen; however, significantly higher scores of IA and not H-I symptoms on the AISRS in the SCT+ cohort were observed. It should be noted that the regression analyses conducted on this full sample are consistent with the data analyses suggested in the publication of the interim data (15).

Neurocognitive testing supported and amplified these findings; there was a distinct pattern of EF deficits among individuals with SCT in this sample of adults with ADHD. SCT was associated with difficulty processing information rapidly in the context of increasing cognitive load, and less effective strategies on working memory tasks. These deficits are indicative of problems with planning and strategy use (e.g., set-shifting and maintenance tasks), focus (interference control), and working memory. In this sample, SCT did not associate with simple motor speed or response inhibition above and beyond the deficits already associated with ADHD. Taken together, these findings suggest that SCT represents a phenotype of executive dysfunction that adds additional burden to those EF deficits associated with ADHD. This is consistent with the finding of significantly greater self-reported EF deficits on the BRIEF-A in the SCT+ versus. SCT− cohorts. While it should be noted that this sample was somewhat loaded for EF deficits in requiring a significant score on the BRIEF MCI subscale on enrollment, the majority of participants who met that threshold did not have SCT on a more comprehensive assessment.

This is the first study to perform CANTAB among adults with ADHD + SCT. It is the second study to examine WAIS processing speed among adults with ADHD + SCT (9), and the first to explore the relative contributions of cognitive processing load and psychomotor coordination compared to reaction time in task performance. While some isolated findings on the CANTAB were not confined to SCT, the more specific profile of deficits on the CANTAB converged with self-report and clinician ratings of EF and SCT. The CANTAB findings are of interest, as they illustrate the linkage between neurocognitive performance and behavioral manifestations of EF dysfunction. The objective measures of working memory problems and poor response inhibition manifest in behaviors captured on the clinical reports. Moreover, the objective measures of difficulty with cognitive loads and mental flexibility manifest as difficulty with shifting attention, monitoring behavior during tasks, and flexibly updating mental sets on rating scale reports. The scales, however, may be more meaningful in translating those neuropsychological deficits to problems in life functions and may be more suitable to clinical practice because of testing time and financial demands.

The latent factors elucidated by PAF explain much of the variance traditionally seen in the assessment of ADHD and provide insight into the neurocognitive phenotypes of ADHD and the nature of SCT. All factors show that EF problems are pervasive among adults with ADHD and SCT, and that specific neurocognitive processes may sub-serve the EF problems associated with SCT. The results of the extant literature regarding EF deficits in the context of SCT and ADHD seem to concur. ADHD is more often found through response inhibition, but SCT is more often found in other EF domains [e.g., in studies by Barkley (5), Sergeant (8), and Wood et al. (9)]. In effect, when SCT is added to ADHD, individuals have significantly more issues in processing their environments and responding to task demands especially when cognitive loads are high.

## Limitations

Several factors should be considered when interpreting these data, including the following: (1) weighting the sample toward executive dysfunction by recruiting people who had SCT and a BRIEF MCI of >65; (2) including only adults with ADHD—we are not able to extrapolate these findings to individuals with SCT but without ADHD; and (3) the unbalanced enrollment nature between the two sites. Future studies require larger sample sizes and the inclusion of people without executive function deficits to confirm the deficits reported above, because SCT may be associated with EF deficits, and our recruitment strategy may have a bias toward amplifying this relationship. Notwithstanding these potential limitations, this study highlights the importance of EF deficits, described clinically and neuropsychologically in defining SCT in adults with ADHD; the overlap of clinical and neuropsychological observations reinforces the importance of observing EF deficits in the phenomenology of SCT. Furthermore, the persistence of heightened impairment when controlling for ADHD symptoms highlights the need for further investigations into understanding the impact of SCT in adults.

## Conclusion

To summarize, in this sample of well-characterized adults with ADHD, patient- and clinician-rated cognitive, behavioral, and functional status were objectively supported by neurocognitive performance. These measures were discrete and separable from those usually associated with ADHD. Further, the neurocognitive and clinical reports, when taken together, defined multiple constructs representing cognitive phenotypes. SCT was associated with distinct, measurable, and objective neurocognitive dysfunctions that directly relate to the functional impairments frequently reported by patients. The results of this study extend the scientific basis of SCT as a neurocognitive syndrome within ADHD and provide reassurance that clinically obtained measures are valid and useful when neurocognitive testing is not feasible. For clinical practice, the data suggest that the evaluation of SCT in patients with ADHD can add important information for understanding the patients’ challenges and for treatment planning.

## Data Availability

The authors will make their data available as is necessary, acceptable, and reasonable on request.
