# Supplementary material for: Characteristics of Sluggish Cognitive Tempo among adults with ADHD: objective neurocognitive measures align with self-report of executive function
Source: Front Child Adolesc Psychiatry. 2023 Jul 24;2:1188901. doi: 10.3389/frcha.2023.1188901 (PMC11747905; doi:10.3389/frcha.2023.1188901)
Supplement: Supplementary file 1 [file Datasheet1.docx]

Although this was an exploratory analysis with a relatively small sample, we were unable to retain all factors with Eigenvalue greater than 1. Retention of factors based on the Eigenvalue is an over-inclusive method that retains noise, and due to the high communality between the measures, we were required to constrain the parameters in order to extract meaningful factors. To do this, we examined the accompanying scree plot to find the natural break point. This point was arguably between 3 and 5, so in following Geert van den Berg (1) we examined the 5 factor’s Eigenvalues and contribution to the variance, and we determined that the true break point was after factor 3. The resulting 3 factor pattern (containing the coefficients for the linear combination of the variables) and structure matrices of the PAF were determined to be stable, containing 5 or more contributing items (1).

Factor 1 had an Eigenvalue of 7.5 and accounted for 20.9% of the variance. It was labeled the SCT Factor and showed the associations between SCT, the clinical variables, and EF measures. This was the only factor to include SCT as a predictor on the Pattern Matrix. In all, there were 14 strong components in this grouping. The remaining components were EF problems as reported on all nine subscales of the BRIEF-A; EF problems, ED problems, Hyperactivity/Impulsivity, and Inattention as rated by clinicians on all four domains of the AISRS.

Factor 2 had an Eigenvalue of 4.7 and accounted for 13.1% of the variance. It was labeled the Executive Function (EF) Factor. There were 8 strong components in this grouping: four variables measuring longer response latencies for all task conditions (congruent cue, incongruent cue, and switching block) of the CANTAB AST, a variable reflecting increased cognitive burden during tasks that require mental flexibility (switching cost) on the AST, and three variables of the CANTAB SST task that represent the cognitive burden during response inhibition (faster response times when inhibition is not required (on “go” tasks) within the context of greater variability in response times indicates a higher cognitive cost of inhibition responses).

Factor 3 had an Eigenvalue of 2.6 and accounted for 7.2% of the Variance. It was labeled the Distractibility Factor. Twelve components had strong or moderate loadings on this factor. It is notable that the loading for AISRS ED was .000 for this factor on the Pattern Matrix, as compared to weak but non-zero loadings on the other two. The items most strongly contributing to this factor were negative associations (meaning better) WAIS-5 PSI and performance on its component scales. Other contributors were: longer RTs on the CANTAB SST, with less variability in performance over time (SST SSD); better detectability, but more false alarms, and longer latencies on the CANTAB RVP: longer latencies on the CANTAB MOT: lower percent correct trials on the CANTAB AST: and worse performance on the CANTAB Working Memory task.

Review of the Structure Matrix supported interpretation of the Factor1 and Factor 2 from the Pattern Matrix, and identified expanded items for Factor 3 that account for the moderate correlations between factors 1 and 3, and 2 and 3. Factor 3 shares latency loadings with Factor 2. Factor 3 also shares SCT and three measures of EF (Working Memory, Shift, and Self-Monitor) with Factor 1. On the Structure Matrix, Factor 1 also picked up the loading for CANTAB Working Memory.

1. Costello AB, Osborne J. Best practices in exploratory factor analysis: four recommendations for getting the most from your analysis. Pract Assess Res Evaluation. (2005) 10(1):7. doi: 10.7275/jyj1-4868.
